# Relation between Reactive Surface Sites and Precursor Choice for Area-Selective Atomic Layer Deposition Using Small Molecule Inhibitors

**DOI:** 10.1021/acs.jpcc.1c10816

**Published:** 2022-03-08

**Authors:** Marc J. M. Merkx, Athanasios Angelidis, Alfredo Mameli, Jun Li, Paul C. Lemaire, Kashish Sharma, Dennis M. Hausmann, Wilhelmus M. M. Kessels, Tania E. Sandoval, Adriaan J. M. Mackus

**Affiliations:** † Department of Applied Physics, Eindhoven University of Technology, 5600MB Eindhoven, The Netherlands; ‡ TNO-Holst Centre, 5656 AE Eindhoven, The Netherlands; § Lam Research Corporation, Tualatin, Oregon 97062, United States; ∥ Department of Chemical and Environmental Engineering, Universidad Técnica Federico Santa María, 8940000 Santiago, Chile

## Abstract

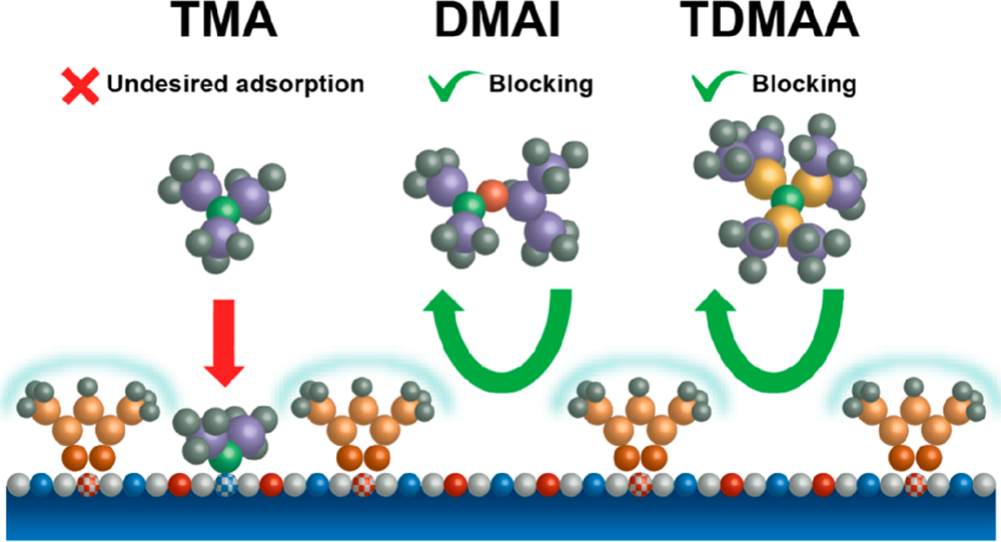

Implementation of vapor/phase dosing of small molecule inhibitors (SMIs) in advanced atomic layer deposition (ALD) cycles is currently being considered for bottom-up fabrication by area-selective ALD. When SMIs are used, it can be challenging to completely block precursor adsorption due to the inhibitor size and the relatively short vapor/phase exposures. Two strategies for precursor blocking are explored: (i) physically covering precursor adsorption sites, i.e., steric shielding, and (ii) eliminating precursor adsorption sites from the surface, i.e., chemical passivation. In this work, it is determined whether steric shielding is enough for effective precursor blocking during area-selective ALD or whether chemical passivation is required as well. At the same time, we address why some ALD precursors are more difficult to block than others. To this end, the blocking of the Al precursor molecules trimethylaluminum (TMA), dimethylaluminum isopropoxide (DMAI), and tris(dimethylamino)aluminum (TDMAA) was studied by using acetylacetone (Hacac) as inhibitor. It was found that DMAI and TDMAA are more easily blocked than TMA because they adsorb on the same surface sites as Hacac, while TMA is also reactive with other surface sites. This work shows that chemical passivation plays a crucial role for precursor blocking in concert with steric shielding. Moreover, the reactivity of the precursor with the surface groups on the non-growth area dictates the effectiveness of blocking precursor adsorption.

## Introduction

The downscaling of integrated circuits in the semiconductor industry requires extremely accurate deposition and patterning of materials.^1^ Feature alignment at the nanometer level during the fabrication of state-of-the-art semiconductor devices has become a bottleneck in the advancement to smaller transistor nodes.^1−3^ As a solution, the use of area-selective deposition (ASD) in self-aligned fabrication schemes is being explored in industry and academia.^4−8^ ASD aims at selective deposition of a material on a patterned substrate, such that growth only occurs on the surfaces where deposition is desired (i.e., the growth area), while the growth is blocked on the rest of the substrate (the non-growth area). As a result, ASD allows for bottom-up and self-aligned deposition with respect to underlying device layers.

In the past decade, atomic layer deposition (ALD) has become a well-established and widely used deposition technique in the semiconductor industry.^9,10^ ALD is based on two or more sequential self-limiting surface reactions, enabling atomic level control over the thickness, combined with excellent uniformity and conformality of the deposited material.^10−12^ ALD is highly sensitive on the reactive sites that terminate the surface, which makes ALD a relevant deposition strategy for ASD.^13^ This surface sensitivity can, in some cases, lead to selective precursor or coreactant adsorption enabling area-selective ALD (often termed inherent selectivity).^14−20^ In general, however, the non-growth area needs to be functionalized by using inhibitor molecules to achieve selectivity. To this end, self-assembled monolayers (SAMs) have been studied as inhibaition layers for area-selective ALD.^21−26^ More recently, small molecule inhibitors (SMIs)^27,28^ that can be applied in the vapor/phase during the ALD process are being investigated for area-selective ALD to better meet the requirements for high-volume manufacturing.^29−33^ In our previous work, area-selective ALD of SiO_2_^34,35^ and WS_2_^29^ has been achieved on SiO_2_ based on the selective adsorption of acetylacetone (Hacac) as SMI on various oxides (e.g., Al_2_O_3_) as non-growth area. Furthermore, we demonstrated area-selective ALD of TiN on SiO_2_ and Al_2_O_3_ by exploiting selective adsorption of aniline on metallic Co and Ru surfaces.^31^ In addition, Kim et al. reported area-selective Al_2_O_3_ ALD on SiO_2_ exploiting the selective adsorption of ethanethiol on Co and Cu.^32^ Studies by Khan et al. and Soethoudt et al. have also shown that ALD precursors with inherent selectivity for precursor adsorption can be employed as SMI to achieve area-selective ALD.^36,37^

**Figure 1:**
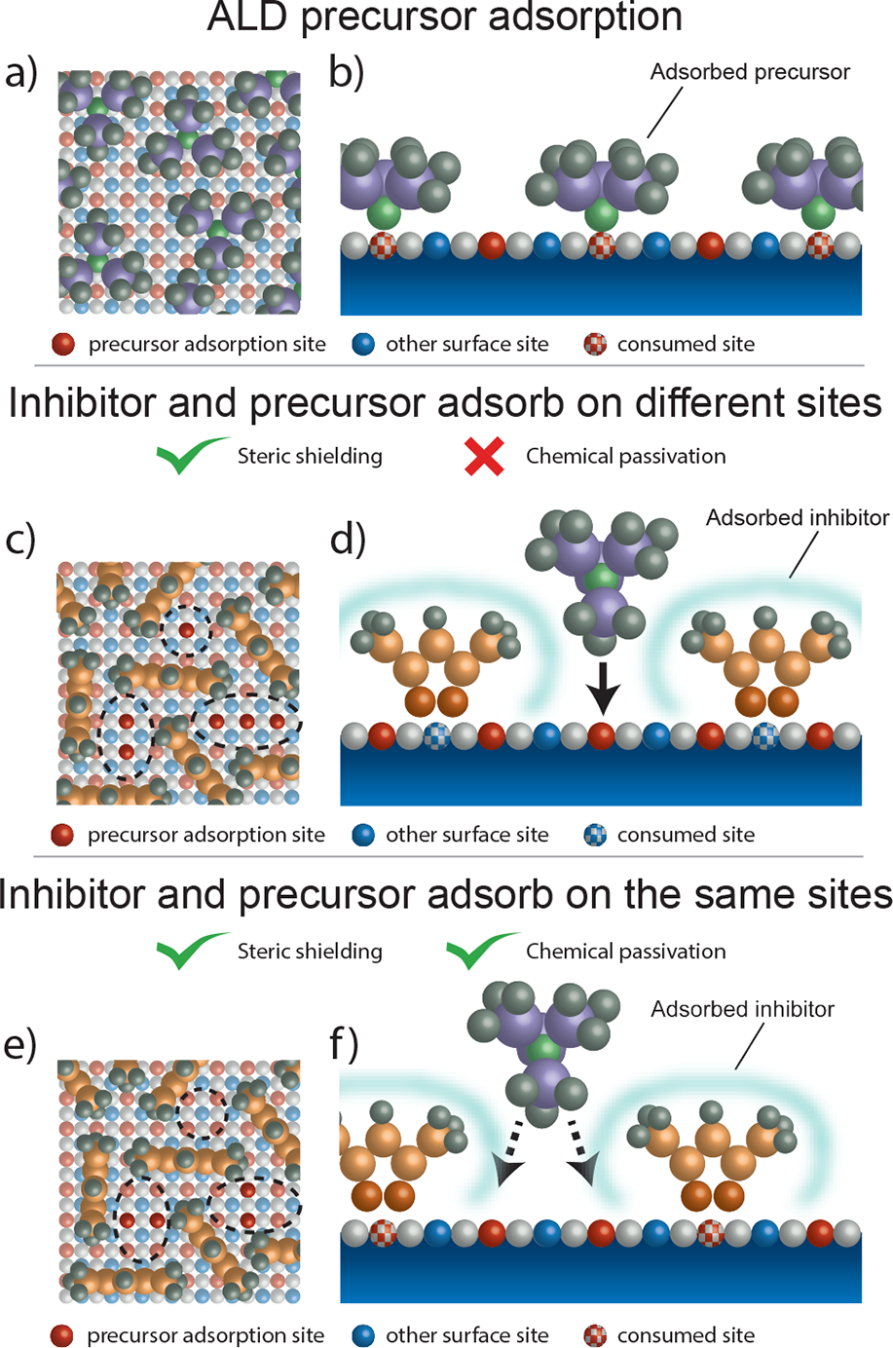
(a, c, e) Top and (b, d, f) side view illustrations showing the two mechanisms (steric shielding and chemical passivation) that contribute to precursor blocking by SMIs. (a, b) Typically not all the surface sites on a material are reactive to the precursor. (c, d) When the inhibitor does not adsorb on the same surface sites as the precursor molecule, precursor blocking can only be achieved through steric shielding. However, because of the lack of surface ordering for SMIs, (c) relatively large gaps can occur in between the inhibitor molecules where the precursor could potentially interact with the surface as indicated by the dashed circles. (e, f) Alternatively, if the inhibitor and precursor adsorb on the same surface sites, the consumption of surface sites as a result of inhibitor adsorption reduces the number of surface sites available for precursor adsorption, here termed chemical passivation, and ensures that the inhibitor sterically covers the right surfaces sites. As a result, the gaps in between the inhibitor molecules do not provide access to precursor adsorption sites in contrast to the situation in (c, d).

When SAMs are used for surface functionalization, the SAM acts as a physical barrier to block precursor adsorption.^38−40^ SAMs block precursor adsorption by physically preventing the precursor from reaching the precursor adsorption sites on the non-growth area, here termed steric shielding. To obtain an effective physical barrier, SAMs are typically formed by using wet chemistry at low temperature. During SAM formation, van der Waals forces between the SAM monomers ensure that a well-ordered and high-density SAM is grown.^41^ Unlike SAMs, SMI adsorption does not lead to a highly ordered inhibitor layer on the non-growth area^39^ since the intermolecular forces between adsorbed SMIs are too small to induce surface ordering.^42^ In addition, because of the vapor/phase delivery of the SMI, inhibitor adsorption takes place successively and randomly on the surface, which typically results in a suboptimal inhibitor coverage, leaving gaps in between adsorbed inhibitors where precursor adsorption could potentially take place (see Figure 1c). Preliminary random sequential adsorption (RSA) simulations (also known as stochastic simulations in the literature)^43−46^ show that these gaps can be up to 0.45 nm in radius for Hacac adsorbed on Al_2_O_3_, whereas, for example, the radius of a trimethylaluminum (TMA) precursor molecule is <0.4 nm in radius.^47^ The lack of surface ordering and random nature of inhibitor adsorption make it challenging to achieve the high surface packing density that is required to physically prevent the precursor from reaching the surface.

As shown in Figure 1a, ALD precursor adsorption is typically not possible on all surface sites but instead depends on the availability of specific surface groups (e.g., hydroxyl groups).^11^ For example, the growth per cycle (GPC) of Al_2_O_3_ ALD using trimethylaluminum (TMA) and O_2_ plasma has been reported to decrease with increasing temperature, which is attributed to the conversion of surface hydroxyl groups to Al–O–Al bridges on which TMA adsorption is less favorable.^48^ Processes based on inherent selectivity exploit this sensitivity by using substrate materials that lack certain precursor adsorption sites or by employing surface pretreatments to remove precursor adsorption sites from the non-growth area.^14,15,49^ In other words, the non-growth area is (made) chemically unreactive for precursor adsorption, here termed chemical passivation. For example, a HF dip can be used to strip the native oxide and OH groups from a Si surface, leaving the surface H-terminated, and thereby, chemically passivated for the adsorption of specific ALD precursors.^14−16^

If the inhibitor adsorbs on the same surface sites as the precursor, the inhibitor can also chemically passivate the non-growth area as shown in Figure 1e,f. This strategy has been discussed in the work of Yanguas-Gil et al. by stating that precursor blocking using SMIs is more effective when there is overlap between the reactive surface sites involved in precursor and inhibitor adsorption.^27^ Adsorption of the inhibitor on the same surface sites as the precursor ensures that the inhibitor molecules sterically cover the right surface sites and typically also consumes surface groups such that they are no longer available for ALD precursor adsorption. Chemisorption of an inhibitor on a surface site often involves the formation of a volatile reaction product which is pumped away after inhibitor dosing; for example, the chemisorption of acetylacetone on an oxide surface results in an acac adsorbate and volatile H_2_O. As a result, the surface sites that the inhibitors react with are not only occupied but also consumed (i.e., removed from the surface). Chemical passivation can, therefore, be employed to supplement physical passivation by reducing the reactive surface sites available on the surface.

Note that the adsorption of an inhibitor molecule typically contributes to both chemical passivation and steric shielding, since the adsorption involves consumption of surface groups as well as shielding of sites. Considering typical surface site densities and inhibitor sizes, the inhibitor molecules are not expected to consume all surface sites on the non-growth area. For example, the Al_2_O_3_ surface used in this work contains ∼7 OH groups/nm^2^,^50,51^ whereas Hacac requires at least ∼0.3 nm^2^/molecule for adsorption based on van der Waals radii of CH_3_, CH_2_, and CO groups in the acac adsorbate.^52,53^ Consequently, Hacac has been reported to adsorb on an Al_2_O_3_ surface with a density of ∼2.1 molecules/nm^2^ in saturation, corresponding to a consumption of only 2.1 OH groups/nm^2^ at most.^54^ To put these numbers into perspective, SAMs formed by using octadecylphosphonic acid (ODPA) are reported to have packing densities of around 4 molecules/nm^2^,^55,56^ which illustrates that SMIs cannot rely on steric shielding as much as SAMs. Overall, a combination of steric shielding and chemical passivation is likely needed to achieve effective precursor blocking for SMIs.

The choice of precursor determines which surface sites need to be chemically passivated by the inhibitors and can therefore play an important role in the selectivity of an area-selective ALD process. The precursor has been reported to strongly affect the selectivity when SAMs are used as inhibitor, e.g., depending on the precursor reactivity and precursor diffusion rates through the SAM.^57−59^ For the SMI ethanethiol, Kim et al. reported that the ALD precursor dimethylaluminum isopropoxide (DMAI) is more easily blocked as opposed to trimethylaluminum (TMA) because DMAI is a dimer at ALD conditions,^32^ whereas for TMA, at least a portion of the precursor molecules is in monomeric form above 70 °C.^60,61^ Although these studies explore various mechanisms for how the precursor affects the selectivity of the process, the interplay between the surface chemistry of the chosen substrate materials (in terms of available surface sites) and the precursor chemistry is not explored so far.

In this work, precursor blocking by SMIs is studied by using in situ infrared (IR) spectroscopy, focusing on the question of whether steric shielding is sufficient for precursor blocking or whether chemical passivation is also required. In addition, the mechanisms that contribute to why some ALD precursors are more easily blocked than others are addressed. To this end, the blocking of trimethylaluminum (TMA), dimethylaluminum isopropoxide (DMAI), and tris(dimethylamino)aluminum (TDMAA) was studied on an Al_2_O_3_ surface by using Hacac as inhibitor. The observed precursor blocking was found to depend strongly on the overlap in surface sites for precursor and inhibitor chemisorption. The results show that precursor choice is vital to consider for area-selective ALD.

## Methods

### Reactor

All experiments were performed on a home-built ALD reactor^30^ equipped with a turbomolecular pump backed up by a roughing pump leading to a base pressure of ∼10^–6^ mbar. Infrared (IR) light can enter and exit the reactor through KBr windows, which are protected from deposition using gate valves. The powder samples are moved into the IR beam by using a Prevac manipulator described in more detail in previous work.^30^

### ALD Precursors and Inhibitor Molecules

Hacac [Sigma-Aldrich, synthesized by Wacker Chemie AG, Burghausen, Germany, ≥99.5% (GC), CAS number 123-54-6] was used as inhibitor. The TMA [Dockweiler Chemicals GmbH, ≥99.9999 metal purity, CAS number 75-24-1], DMAI [Dockweiler Chemicals GmbH, ≥99.999 metal purity, CAS number 6063-89-4], and TDMAA [Sigma-Aldrich, CAS number 32093-39-3] precursors were vapor-drawn into the reactor by using a bubbler temperature of 20, 60, and 90 °C, respectively. The inhibitor and precursor molecules were dosed with a gate valve to the pump closed, keeping the molecule trapped in the reactor for 10 s before pumping down. Pulse durations of 5 s, 100 ms, 1 s, and 2.5 s were used for dosing Hacac, TMA, DMAI, and TDMAA, respectively. These pulses were repeated until no more inhibitor or precursor adsorption was detected by using IR spectroscopy. Saturation was typically reached around 50 s, 900 ms, 30 s, and 45 s for Hacac, TMA, DMAI, and TDMAA, respectively.

### Infrared (IR) Spectroscopy

The in situ IR spectroscopy measurements were performed according to the procedure and setup reported in previous work.^30^ In short, a nonporous AEROSIL OX 50 SiO_2_ powder substrate is used that is pressed into a tungsten mesh (Alfa Aesar) through which a current can be send for heating. A thermocouple is welded to the tungsten mesh to monitor its temperature in situ. Prior to the experiment, the substrate is coated with Al_2_O_3_ by using 30 ALD cycles of TMA and H_2_O at ∼300 °C. Because the exact amount of powder that was pressed into the mesh cannot be controlled, a single cycle of TMA/H_2_O is measured by using IR spectroscopy after coating the substrate such that the accessible powder area can be normalized for sample-to-sample comparisons.^30^ Note that this normalization procedure makes the noise appear larger for data measured on samples with a relatively small amount of accessible powder area. For each IR spectrum, 1024 intensity scans were averaged, corresponding to ∼2 min per IR measurement. After each inhibitor or precursor dose, the reactor was pumped down to 10^–5^ mbar (10–30 s) before starting the IR measurement to avoid gas-phase species from affecting the spectrum. Unless stated otherwise, precursor blocking was studied at 150 °C.

### Density Functional Theory (DFT) Calculations

The level of theory used for the DFT calculations is discussed in detail in a previous work.^30^ In summary, we calculated electronic energies using the Vienna Ab-Initio Simulation Package (VASP) 5.4.4,^62−65^ as implemented in MedeA-VASP software package.^66^ Hacac adsorbed on Al_2_O_3_ was calculated by using the generalized gradient approximation (GGA) functional by Perdew, Burke, and Ernzerhof (PBE)^67,68^ with the dispersion correction D3 and the Becke–Johnson (BJ) damping function.^69,70^ For convergence, the projector augmented wave formalism (PAW)^71,72^ with a plane wave cutoff of 400 eV was used. The self-consistent field (SCF) cycle was converged with an accuracy of 10^–5^ eV and the geometry was optimized up to 10^–2^ eV Å^–1^. The Al_2_O_3_ surface was created by using a four-layer 3 × 3 supercell of partially hydroxylated α-Al_2_O_3_(0001). It should be noted that unlike the amorphous Al_2_O_3_ surface used in the IR experiments, the modeled surface is crystalline and does not contain vicinal OH groups due to computational limitations. The Brillouin zone was sampled by a Gamma-centered 2 × 2 × 1 Monkhorst–Pack grid;^73^ a Gaussian smearing of 0.1 eV and a gap of 17 Å of vacuum gap in the *z*-direction were used to accommodate the Hacac molecule. The bottom three layers of atoms were kept fixed, and the remaining atoms of the slab and Hacac were relaxed.

## Results

**Figure 2:**
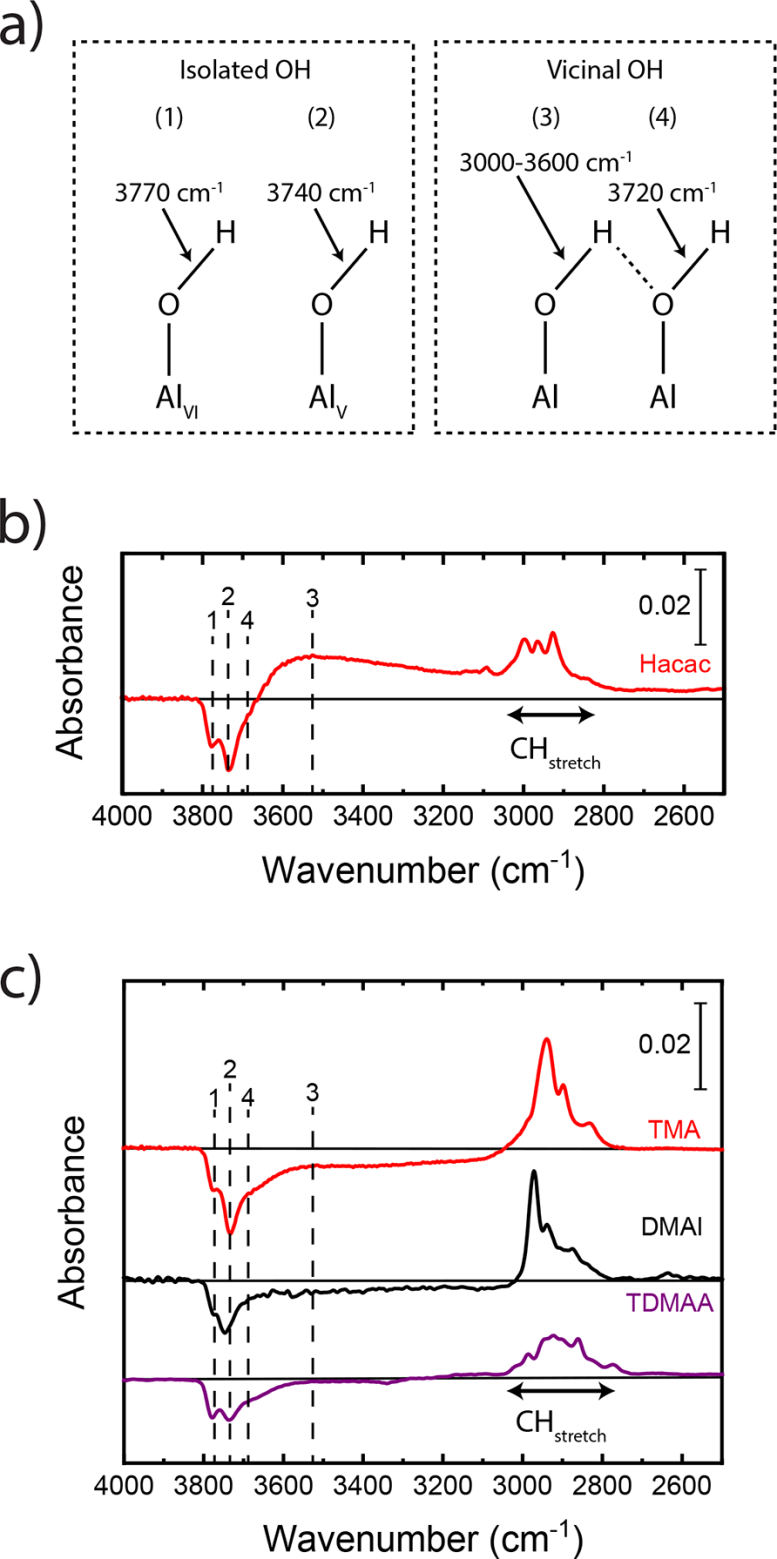
(a) Schematic illustrations of isolated and vicinal hydroxyl surface groups on an Al_2_O_3_ surface with the wavenumbers corresponding to their OH stretching vibrations.^50,75,76^ The hydroxyl groups for Al atoms coordinated to six (i.e., Al_VI_) or five O atoms (i.e., Al_V_) are shown. Two kinds of vicinal OH groups exist on the surface depending on whether the H (i.e., 3) or O (i.e., 4) atom in the OH group is hydrogen bonded. IR difference spectra showing the consumption of hydroxyl groups after (b) Hacac inhibitor adsorption and after (c) TMA, DMAI, and TDMAA precursor adsorption. The numbered dashed lines in the IR spectra correspond to the schematic illustrations in (a). Inhibitor and precursor adsorption was performed at 150 °C.

The surface of a metal oxide typically contains several different types of OH groups, e.g., isolated OH groups and hydrogen bonded (i.e., vicinal) OH groups (see Figure 2a).^51,74,75^ These OH groups are not necessarily all involved in the chemisorption of the inhibitor and precursor molecules. Figure 2 shows the adsorption of the Hacac inhibitor and several ALD precursor molecules at 150 °C on an Al_2_O_3_ surface as measured by using IR spectroscopy. The IR peaks attributed to the consumption of surface OH groups are indicated (peaks 1–4) in the IR spectra, and the corresponding OH groups are illustrated in Figure 2a. As shown in Figure 2b, the Hacac inhibitor molecules were found to adsorb exclusively on the isolated OH groups (peaks 1 and 2; 3800–3730 cm^–1^) of an Al_2_O_3_ surface. The adsorption of Hacac on an Al_2_O_3_ surface was previously studied by using density functional theory (DFT).^30,34^ These studies show that Hacac forms its most stable adsorption configuration, i.e., the chelate configuration, by transferring a hydrogen atom to a surface OH group and forming H_2_O as volatile reaction product, thereby consuming the surface OH group. Hacac is therefore able to chemically passivate the isolated OH groups on a Al_2_O_3_ surface. Interestingly, the IR spectrum also shows a positive feature in the OH region (peak 3) after Hacac adsorption. This positive feature is attributed to the formation of vicinal OH groups on the Al_2_O_3_ surface as a result of Hacac adsorption. The possible mechanisms that cause these new vicinal OH groups are discussed below.

The IR spectra measured after TMA, DMAI, and TDMAA adsorption on an Al_2_O_3_ surface are shown in Figure 2c. It is clear from those spectra that depending on the choice of ALD precursor, different OH groups can be involved in the chemisorption of the precursor. TMA is observed to adsorb on both isolated (peaks 1 and 2; 3800–3730 cm^–1^) and vicinal (peaks 3 and 4; 3730–3000 cm^–1^) OH groups. DMAI and TDMAA show a much lower reactivity with vicinal OH groups (peaks 3 and 4) and seem to mostly adsorb on isolated OH groups (peaks 1 and 2). For DMAI, the consumption of vicinal OH groups is observed to be 80% smaller as compared to TMA, while TDMAA does not react with vicinal OH groups at all. This observed difference in reactivity with isolated and vicinal OH groups is consistent with previous studies^77−79^ that show a dependence on the molecule for the reactivity to isolated and vicinal OH groups. This difference likely arises from a lower bond dissociation energy for TMA with respect to DMAI and TDMAA,^80^ resulting in a lower energy barrier for the ligand exchange reaction. Correspondingly, for TMA a reaction with vicinal OH groups is kinetically accessible at the experimental conditions, in contrast to DMAI and TDMAA.

**Figure 3:**
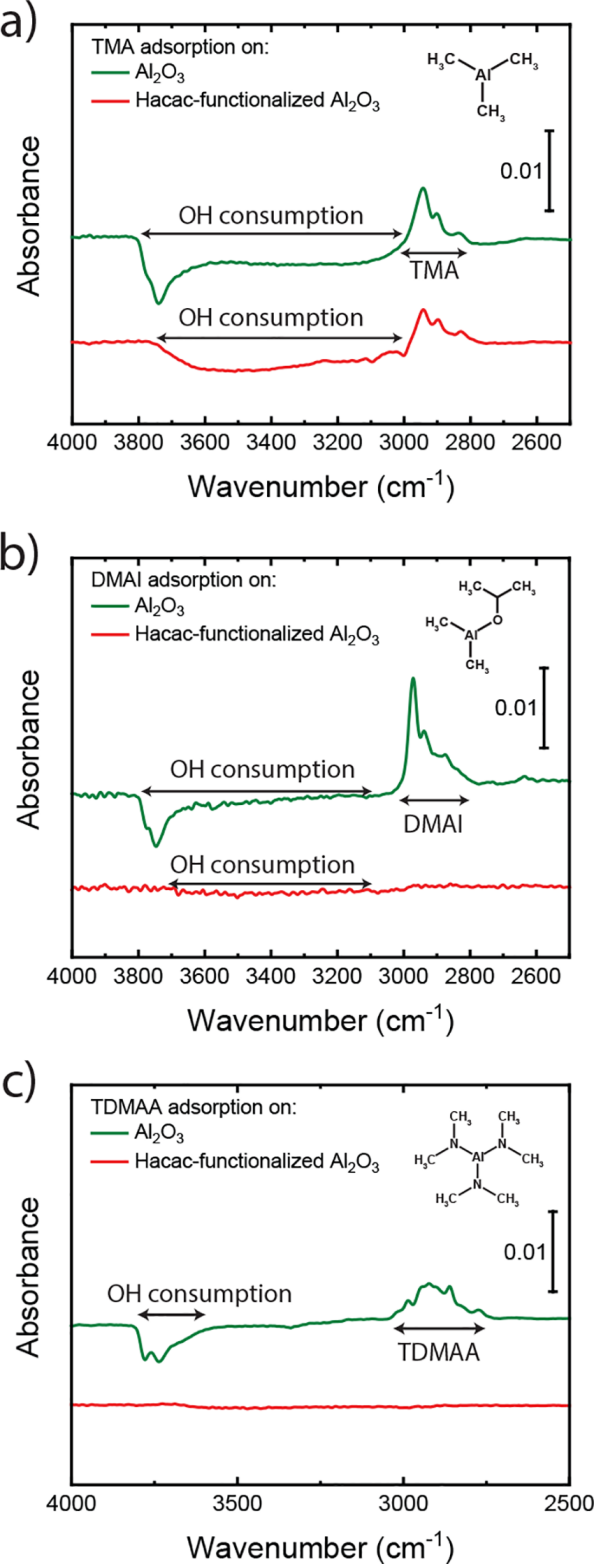
IR difference spectra showing precursor adsorption (or the lack thereof) at 150 °C on a clean Al_2_O_3_ surface and on an Al_2_O_3_ surface that was functionalized with Hacac inhibitor molecules when dosing (a) TMA, (b) DMAI, or (c) TDMAA as the precursor.

The blocking of TMA, DMAI, and TDMAA precursors was studied by using Hacac as inhibitor, as shown in Figure 3. The results show that only roughly one-third of the TMA adsorption is blocked by the Hacac with respect to a nonfunctionalized (i.e., without inhibitor) Al_2_O_3_ surface, which means that the selectivity is already decreased to 0.2 after the first precursor dose. In addition, the consumption of OH groups is clearly observed after TMA adsorption on the Hacac-functionalized Al_2_O_3_ surface, as indicated in Figure 3a. This observed consumption of OH groups was found to consist solely of vicinal OH (i.e., the OH groups on which Hacac does not adsorb) and has a similar amplitude for the IR peak as for vicinal OH consumption when dosing TMA on a clean Al_2_O_3_ surface (see Figure S1). For DMAI, small features in the FTIR spectra could be an indication of the consumption of vicinal OH groups and the adsorption of DMAI; however, these peaks to not exceed the noise level (see Figure 3b). Integration of the CH region indicates that the blocking efficiency of DMAI by Hacac is at least higher than 98%. As shown in Figure 3c, no precursor adsorption or OH group consumption was observed when dosing TDMAA on an Hacac-functionalized Al_2_O_3_ surface. Therefore, the blocking of DMAI and TDMAA adsorption by using Hacac as inhibitor is much more effective as compared to blocking TMA adsorption. These results clearly demonstrate that a good overlap in reactive surface sites for inhibitor and precursor adsorption is beneficial for precursor blocking. Analogously to our previous work,^30^ the studied ALD precursors were found to displace some Hacac molecules from the surface during the precursor dose. Inhibitor displacement leads to a decrease in the inhibitor coverage and therefore to less effective steric shielding by the inhibitor.

**Figure 4:**
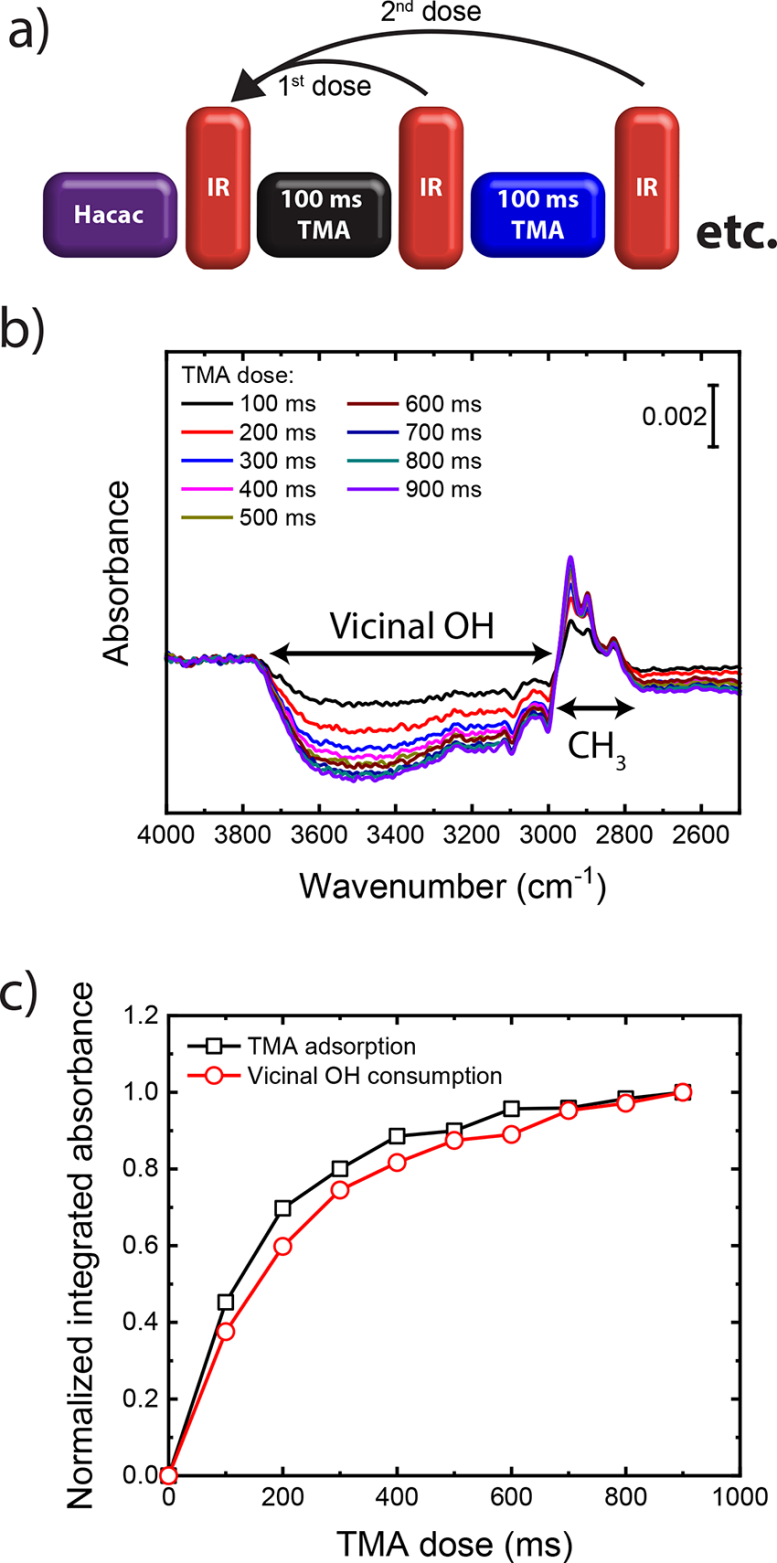
(a) Adsorption of TMA and consumption of vicinal OH groups as a function of TMA dosing (using 100 ms TMA pulses) on an Hacac-functionalized Al_2_O_3_ surface as measured by IR spectroscopy. (b) IR difference spectra using the Hacac-functionalized Al_2_O_3_ surface as reference; see part a. (c) Integrated IR absorbance for the consumption of vicinal OH groups (3800–3250 cm^–1^) and adsorption of TMA (3000–2750 cm^–1^), both normalized to their value at 900 ms TMA dosing. Note that the consumption of OH groups (i.e., negative IR peaks) in (c) is shown as a positive trend due to the normalization of the peak area.

The consumption of vicinal OH groups was found to correlate with the adsorption of TMA on the Hacac-functionalized Al_2_O_3_ surface, as shown in Figure 4. The IR features indicating TMA adsorption increase as a function of TMA dosing (see Figure 4b). The negative IR features that indicate the consumption of vicinal OH groups increase in amplitude as well. Integration of these IR features, shown in Figure 4c, reveals that the number of adsorbing TMA molecules and the consumption of vicinal OH groups behave similarly as a function of the TMA dose. Overall, the IR spectra suggest that the steric shielding by the adsorbed Hacac is insufficient to prevent TMA adsorption on the vicinal OH groups in between the Hacac molecules.

The role that the vicinal OH groups play in the incomplete blocking of TMA by Hacac was further studied by measuring TMA blocking as a function of temperature (see Figures S2 and S3). The OH density on an Al_2_O_3_ surface decreases with increasing temperature,^81^ but more importantly, the fraction of vicinal OH groups on the surface decreases (see Figure S2). As a result, there are fewer surface sites at higher temperature on which TMA adsorbs but Hacac does not. In other words, chemical passivation is more effective at higher temperatures for blocking TMA adsorption using Hacac. As shown in Figure S3, the reduction in vicinal OH groups at higher temperatures was found to lead to a greatly improved blocking efficiency. At a substrate temperature of 100 °C, only ∼20% of TMA adsorption is blocked by the Hacac molecules, whereas at 250 °C, ∼45% TMA adsorption can be blocked.

**Figure 5:**
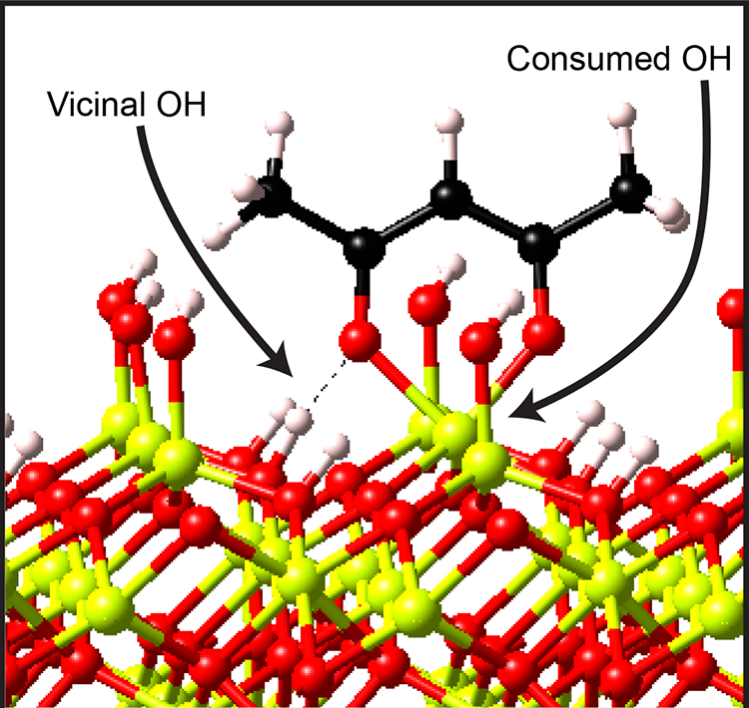
Illustration based on DFT calculations for Hacac adsorption on an Al_2_O_3_ surface. Hacac chemisorption results in the consumption of an OH group through the formation of volatile H_2_O. The original position of this OH is indicated as a consumed OH. Aside from OH group consumption, H bonds can be formed between surface OH groups and Hacac as a result of inhibitor adsorption, which makes the OH groups less reactive for precursor adsorption.

The positive feature in the OH region observed in Figure 2b requires more attention. The formation of new vicinal OH groups as a result of Hacac adsorption could be attributed to (i) physisorbed H_2_O formed as reaction product during Hacac chemisorption and (ii) the formation of hydrogen bonds between isolated OH groups and adsorbed Hacac species. Previous DFT studies show that the H_2_O, which is formed as reaction product, could remain physisorbed with at least 0.25 eV on the Al_2_O_3_ surface after Hacac chemisorption.^34^ However, the IR spectra in Figure 3b,c show no features that indicate a reaction between dosed DMAI and TDMAA with any physisorbed H_2_O that is potentially on the surface, despite the fact that these precursors can normally react with H_2_O as coreactant during ALD.^82,83^ Therefore, either there is no H_2_O on the surface or the H_2_O interacts strongly enough with the surface to prevent reactions with the incoming precursors, in which case the H_2_O actually helps improve the surface coverage and stability of the inhibitor layer. DFT calculations reveal that the observed IR feature in the OH region could also be explained by interactions between isolated OH groups and chemisorbed Hacac (i.e., mechanism ii). These interactions result in the formation of vicinal OH groups from isolated OH groups, as shown in Figure 5. Therefore, the Hacac does not only consume the OH group it chemisorbs on but also can interact with neighboring OH groups, making them less reactive for precursor adsorption.^84,85^ In case the ALD precursor does not react with these vicinal OH groups (e.g., for DMAI and TDMAA), this mechanism contributes to the chemical passivation by the inhibitor. Considering that inhibitor chemisorption cannot take place on every surface site (due to steric limitations), such a mechanism is desirable because it increases the maximum number of surface sites that can be passivated.

## Conclusions

On the basis of the obtained insights, it is possible to distill several requirements for precursor blocking when using SMIs. Steric shielding requires a high surface packing density to block all precursor adsorption. However, because of a lack of surface ordering for SMIs, relatively large gaps can occur in the inhibitor layer (see Figure 1c) which are detrimental for precursor blocking. Alternatively, the non-growth area can be chemically passivated, but this requires that the inhibitor reacts with all surface sites, which is typically very challenging due to steric effects. The Al_2_O_3_ non-growth area studied in this work contains around 7 OH groups per nm^2^ on the surface,^50,51^ meaning that even for an inhibitor with the size of a single methyl group (2 Å van der Waals radius, maximum density of 7.2 groups/nm^2^ assuming hexagonal close packing), it is in practice impossible to chemisorb on all surface OH groups. Interestingly, the IR spectra on TMA blocking (see Figure S1) suggest that two out of three OH groups on the studied Al_2_O_3_ surfaces are vicinal OH groups, meaning that completely blocking the isolated OH groups is much easier as it only requires ∼2 inhibitor molecules per nm^2^. Overall, using a combination of steric shielding and chemical passivation appears to be the best strategy to reach a high selectivity.

Precursor blocking using SMIs strongly depends on the choice of the precursor. The results suggest that a good overlap in the surface sites reactive to inhibitor and precursor adsorption is crucial for obtaining a high degree of precursor blocking. It was found that blocking TMA adsorption is much more challenging as opposed to blocking DMAI and TDMAA adsorption. These observations provide insight into the mechanisms that could be exploited for the development of area-selective ALD processes with a high selectivity. Although TMA is the most used precursor for Al_2_O_3_ ALD, it is likely too reactive to be a suitable precursor for area-selective ALD.

This work describes the role that reactive surface sites play in the challenge of obtaining area-selective ALD with a high selectivity. Typically, selectivity is lost by adsorption of precursor molecules in the gaps in between adsorbed inhibitor molecules as well as by a loss of inhibition. With respect to the latter, inhibitor displacement was observed during the precursor dose, which will be explored in future work. The chemical and physical properties of the employed precursor (e.g., possible adsorption sites, interactions with the inhibitor, and precursor size) significantly affect the selectivity of an area-selective ALD process. Careful selection of the ALD precursor, or even precursor design, is therefore crucial for achieving area-selective ALD with a high selectivity.

## Supporting Information Available

IR spectra comparing the consumption of vicinal OH groups for TMA adsorption on Al_2_O_3_ and Hacac-functionalized Al_2_O_3_; consumption of isolated and vicinal OH groups after TMA chemisorption as a function of temperature; blocking of TMA adsorption by Hacac as a function of TMA adsorption (PDF)
