# Supplementary material for: Relation between Reactive Surface Sites and Precursor Choice for Area-Selective Atomic Layer Deposition Using Small Molecule Inhibitors
Source: J Phys Chem C Nanomater Interfaces. 2022 Mar 8;126(10):4845–53. doi: 10.1021/acs.jpcc.1c10816 (PMC8935369; doi:10.1021/acs.jpcc.1c10816)
Supplement: Supplementary file 1 — jp1c10816_si_001.pdf [file jp1c10816_si_001.pdf]

Supporting information for:

The Relation Between Reactive Surface Sites and Precursor Choice for Area-Selective Atomic Layer Deposition Using Small Molecule Inhibitors

Marc J.M. Merkx,[a] Athanasios Angelidis,[a] Alfredo Mameli,[b] Jun Li,[a] Paul C. Lemaire,[c] Kashish Sharma,[c] Dennis M. Hausmann,[c] Wilhelmus M.M. Kessels,[a] Tania E. Sandoval[d] and Adriaan J.M. Mackus[a]

[a] *Department of Applied Physics, Eindhoven University of Technology, Eindhoven, The Netherlands*

[b] *TNO-Holst Centre, Eindhoven, The Netherlands*

[c] *Lam Research Corporation, Tualatin, Oregon, USA*

[d] *Department of Chemical and Environmental Engineering, Universidad Técnica Federico Santa María, Santiago, Chile*

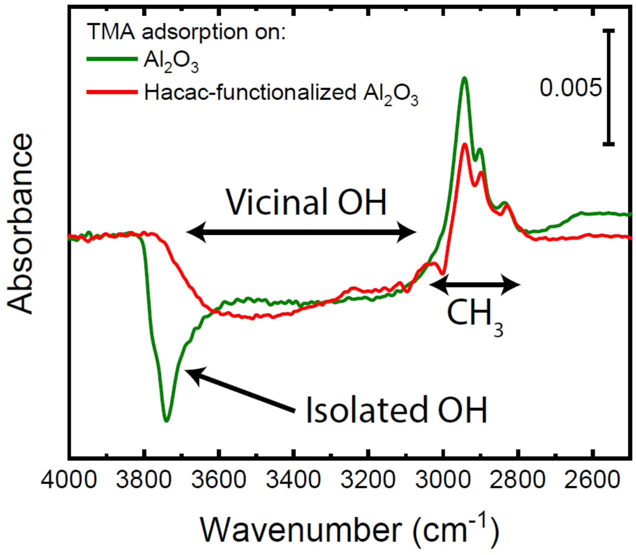

Figure S1: IR difference spectra showing TMA adsorption on a clean Al_2O_3 surface and on an Hacac-functionalized Al_2O_3 surface. The spectra show that, while TMA adsorption consumes isolated and vicinal OH groups on a clean Al_2O_3 surface, only the consumption of vicinal OH groups is detected on the Hacac-functionalized Al_2O_3 surface. The peak indicating the consumption of vicinal OH groups is shown to have a similar amplitude for both spectra. The amplitudes of the CH_3 stretching mode for TMA adsorption on both cases suggests that 2/3 of the OH groups on the Al_2O_3 surface are vicinal OH groups.

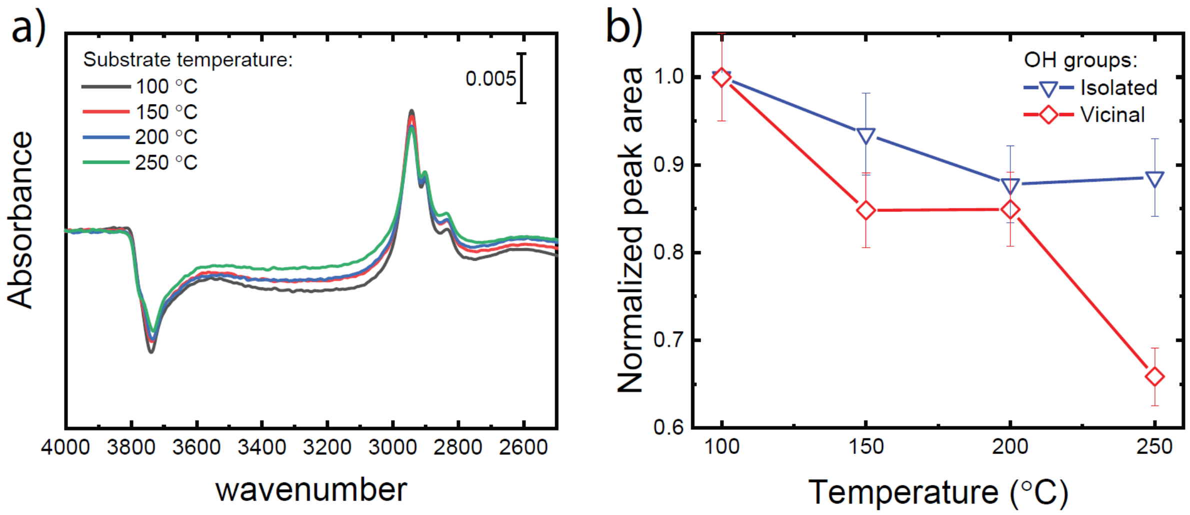

Figure S2: TMA adsorption on an Al_2O_3 surface as function of temperature. (a) IR difference spectra for TMA adsorption at different substrate temperatures. (b) IR peak area for the isolated and vicinal OH group consumption observed in (a). The peak area is normalized to the value at 100 °C.

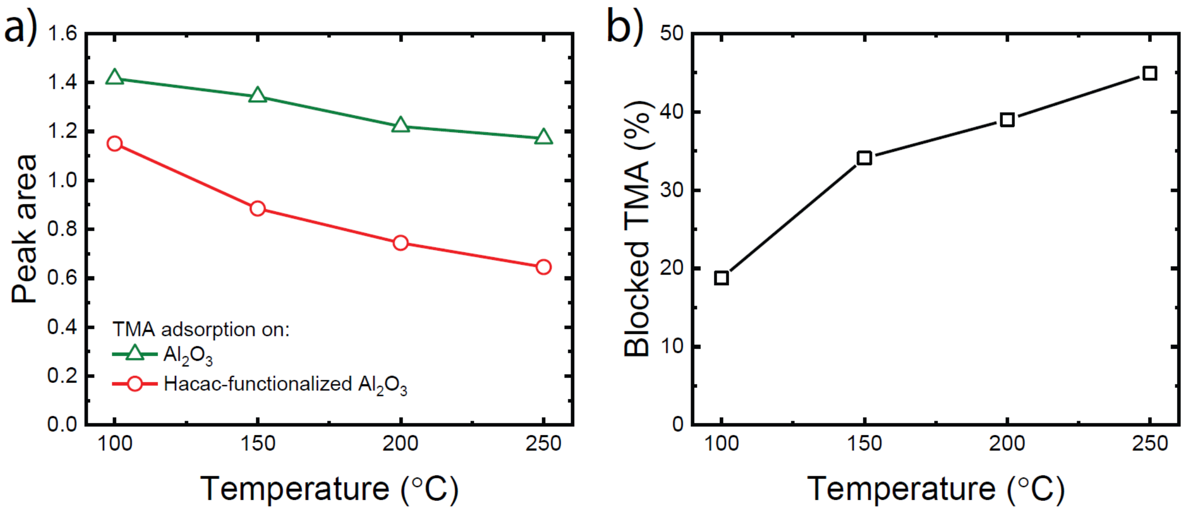

Figure S3: Blocking of TMA adsorption by Hacac inhibitor molecules as a function of temperature. (a) TMA peak area as measured by IR spectroscopy for TMA adsorption on a clean and Hacac-functionalized Al_2O_3 surface. (b) Percentage of the TMA adsorption that is blocked on the Hacac-functionalized surface with respect to TMA adsorption on a clean Al_2O_3 surface as a function of temperature. Unfortunately, Hacac dissociatively desorbs at temperatures above 250 °C,[1] but potentially a high temperature pretreatment of the surface could be employed to improve precursor blocking for TMA further.

Bibliography:

(1) Kytokivi, A.; Rautiainen, A.; Root, A. *J. Chem. Soc. Faraday Trans.* **1997**, *93*, 4079–4084.
